# Using a knowledge translation framework to identify health care professionals’ perceived barriers and enablers for personalised severe asthma care

**DOI:** 10.1371/journal.pone.0269038

**Published:** 2022-06-07

**Authors:** Eleanor C. Majellano, Vanessa L. Clark, Rebecca F. McLoughlin, Peter G. Gibson, Vanessa M. McDonald

**Affiliations:** 1 National Health and Medical Research Council Centre for Research Excellence in Severe Asthma and The Priority Research Centre for Healthy Lungs, The University of Newcastle, Newcastle, New South Wales, Australia; 2 School of Nursing and Midwifery, The University of Newcastle, Newcastle, New South Wales, Australia; 3 National Health and Medical Research Council Centre for Research Excellence in Treatable Traits, New Lambton Heights, New South Wales, Australia; 4 Department of Respiratory and Sleep Medicine, John Hunter Hospital, Hunter Medical Research Institute, Newcastle, New South Wales, Australia; Boston Children’s Hospital, UNITED STATES

## Abstract

**Background:**

Whilst multidimensional assessment enables the detection of treatable traits in severe asthma and has the potential to improve patient outcomes, healthcare disparities exist, and little is known about the factors influencing optimal management in severe asthma. This study aimed to explore perceived barriers, and enablers to implementing personalised care in severe asthma, from the healthcare professionals’ perspective.

**Methods:**

A descriptive, qualitative study involving a single focus group (n = 7) and semi-structured interviews (n = 33) with multidisciplinary healthcare professionals involved in severe asthma care was conducted. A hybrid thematic and content analysis was undertaken to identify themes, which were then deductively mapped to the Theoretical Domains Framework (TDF).

**Results:**

Overall, three emergent themes were identified: (1) Barriers- (2) Enablers- to optimal management; (3) Desired model of care. Across all TDF domains, 6 constructs influenced development and implementation of optimal care: (1) belief about consequences, (2) environmental context and resources, (3) belief about capabilities, (4) social/professional role and identity, (5) goals and (6) knowledge.

**Conclusion:**

Implementation of personalised care in severe asthma is complex and non-linear. The use of a theory-based approach effectively demonstrated how a variety of behaviours could be targeted to optimise and promote personalised care in different clinical setting.

## 1 Introduction

Meeting the healthcare needs of people with severe asthma requires comprehensive and personalised approaches (individualised therapy based on genetic, biomarker, phenotypic or psychological features that distinguishes a specific patient from other individuals with comparable presentations) [1] involving multidisciplinary teams (MDT) [2]. Whilst severe asthma guidelines recommend systematic/multidimensional assessment to improve health-related quality of life and asthma control and to reduce acute attacks, the translation of this approach to practice remains a challenge [3, 4]. Furthermore, fragmented care has been reported by clinicians involved in severe asthma management, leading to inconsistent and delayed referral and access to specialist care [5, 6]. Healthcare professional (HCP)-related barriers have been identified and reported for several areas of asthma management [7, 8]. Among these barriers are a lack of familiarity with guidelines [9], clarity of role [10] and poor communication [11]. The limitation of these studies, from a severe asthma perspective is that these report HCPs’ perceptions of only general asthma care rather than their views or beliefs related to severe asthma management. As severe asthma presents as a different disease in terms of heterogeneity and complexity it would be erroneous to extrapolate these data and apply them in the severe asthma setting. Therefore, attention to factors that impede HCPs in implementing optimal management of severe asthma is an immediate priority.

Effective implementation of personalised care in severe asthma requires an understanding of determinants that influence behaviour change [12]. The Theoretical Domains Framework (TDF) has been proposed as a mechanism to understand the factors that influence HCP behavioural change [12] and has been used in different clinical areas [13–16]. The TDF comprises 14 domains, which can be used to explore influencing factors and support tailored interventions [17]. This study aims to identify and describe HCPs’ perceived barriers and enablers to implementing personalised care in severe asthma through in-depth qualitative interviews and a focus group. Determinants were then mapped to the domains of the TDF to understand management behaviours and propose a model of care for severe asthma.

## 2 Methods

### 2.1 Design

We conducted a descriptive qualitative inquiry, combining a focus group and semi-structured interviews with multidisciplinary HCPs involved in the care of severe asthma patients. The Consolidated Criteria for Reporting Qualitative Research (COREQ) [18] was used to guide reporting (S1 Table in S1 File). Ethics approval was obtained from the Hunter New England Health Human Research Ethics Committee 2019/ETHO0143/H-2019-0208.

### 2.2 Participants and recruitment

HCPs were selected using a stratified purposive sampling strategy to ensure heterogeneity across professions, organisations and experience. Inclusion and exclusion criteria are provided in Table 1.

**Table 1:** Inclusion and exclusion criteria.

Inclusion	Exclusion
• Able to provide written informed or digital consent	• Practicing clinicians outside Australia
• ≥ 18 years of age	
• English-speaking	
• Australian Health Practitioner Regulation Agency–registered	
• Practicing multidisciplinary healthcare professionals within Australia with experience in providing care and management of people with severe asthma	

HCPs from the Severe Asthma Clinic of John Hunter Hospital (JHH) were invited to participate in a focus group. When interested participants were unable to attend, individual interviews were offered. Interview participants were recruited from the Australasian Severe Asthma Registry (ASAR), Australian Mepolizumab Registry, The Thoracic Society of Australia and New Zealand (TSANZ), and professional networks. Study invitations were sent via email, the intranet or newsletters enclosing the study information and consent form. All interested participants directly contacted the researcher to indicate their interest and written or digital consent was gained prior to the focus group or interview.

### 2.3 Data collection

Recruitment took place between September 2019 and June 2020. A single focus group (n = 7) was conducted by trained and experienced qualitative researchers (EM and VC). The focus group took place in a private room at the Hunter Medical Research Institute (HMRI) and its duration was 50 minutes. All in-depth interviews (n = 33) were conducted by EM, either face-to-face or via teleconference, in a private room at the JHH/HMRI. Interview duration ranged from 13–49 (mean 26, SD 10) minutes. The focus group and all interviews were guided by the same interview schedule developed around the study aims and a literature review [2, 3, 5, 6, 19–30] (S2 Table in S1 File), and were digitally recorded with field notes. All participants were asked what design or ideal model of care they would want to have in severe asthma. They were asked to either draw or describe descriptively what the model should look like. The focus group participants were given a sheet of paper to outline the elements of their model. In-depth interviews were conducted until reaching data saturation. This was established when no new codes are identified or when the participants’ perspectives became recurrent and corresponded to previously obtained data. There were no relationships established between the participants and the interviewer (EM) prior to the study. No incentives were given, nor repeat interviews conducted. Interviews were conducted until no new themes emerged.

### 2.4 Data analysis

**Fig 1:**
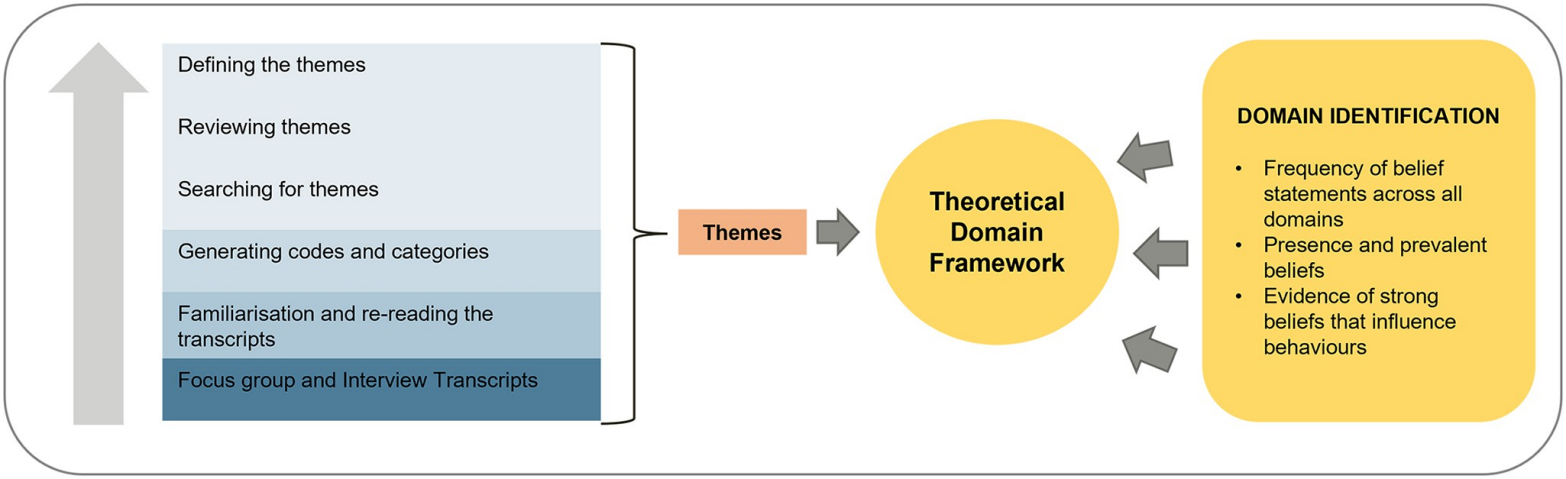
The two-stage hybrid inductive and deductive analysis. First, themes were inductively identified. This began with familiarisation with and re-reading the transcripts, followed by an initial coding and grouping of common codes into emergent themes. Next, a deductive approach was used to link the naturally occurring themes to domains of the Theoretical Domains Framework.

Focus group and interview data were combined for the purpose of analysis. Digital interview recordings were deidentified and transcribed verbatim by professional transcription services via a secure encrypted cloud service. NVivo Pro version 12 software was used to aid data management. Transcripts were verified against the original sound file by EM for accuracy. Data analysis followed a two-stage process combining thematic analysis and frequency content analysis (Fig 1).

Firstly, an inductive thematic approach [31] was used to identify the themes. Codes and emerging themes were then reviewed and discussed with co-investigators (VM and VC) with qualitative research expertise. Major themes and subthemes were refined and grouped until consensus was obtained between co-authors. Secondly, we undertook a deductive approach whereby we linked the themes to the TDF domains. We followed the recommended procedure for TDF analysis [32] to identify the relevant TDF domains that were most likely to influence HCP behaviour/beliefs (Fig 1). To facilitate visual interpretability, descriptive statistics were used to summarise the frequency of belief statements across TDF domains. Transcripts were frequently revisited throughout the analysis to confirm the consistency of themes.

## 3 Results

### 3.1 Participants

Table 2 describes participant characteristics.

**Table 2:** Characteristic of participants.

Characteristic	Total participants N (%) n = 40	Focus groups N (%) n = 7	Individual interviews N (%) n = 33
**Sex**			
Male	18 (45)	1 (14)	17 (52)
Female	22 (55)	6 (86)	16 (49)
**State**			
New South Wales	29 (72)	7 (100)	22 (67)
Queensland	7 (17)	0 (0)	7 (21)
Victoria	1 (3)	0 (0)	1 (3)
South Australia	2 (5)	0 (0)	2 (6)
Western Australia	1 (3)	0 (0)	1 (3)
**Location of medical practice**			
Metro	37 (93)	7 (100)	30 (91)
Regional	3 (7)	0 (0)	3 (9)
**Medical profession**			
General practitioner	2 (5)	0 (0)	2 (6)
Respiratory specialist*	10 (25)	0 (0)	10 (30)
Respiratory advanced trainee**	2 (5)	1 (14)	1 (3)
Emergency department specialist	6 (15)	0 (0)	6 (18)
Nurse	16 (40)	4 (57)	12 (36)
Physiotherapist	2 (5)	2 (29)	0 (0)
Speech pathologist	1 (3)	0 (0)	1 (3)
Pharmacist	1 (3)	0 (0)	1 (3)
**Years working in profession**			
< 5	6 (15)	1 (14.3)	5 (15.2)
5–10	10 (25)	1 (14.3)	9 (27.3)
10–15	8 (20)	4 (57.1)	4 (12.1)
15–20	8(20)	0 (0)	8 (24.2)
20–30	3 (7)	1 (14.3)	2 (6.1)
> 30	5 (12)	0 (0)	5 (15.2)
**Current work setting**			
University-affiliated public hospital with a specialist severe asthma clinic	32 (80)	7 (100)	25 (76)
Private and university-affiliated public hospitals with a specialist severe asthma clinic	3 (7)	0 (0)	3 (9)
Private practice	5 (12)	0 (0)	5 (15)

Note:

* a medical professional who specialises in diagnosing, treating and preventing conditions and diseases affecting the respiratory system.

** is a doctor who require 3 years of full-time equivalent respiratory medicine training to receive fellowship of the Royal Australasian College of Physicians.

A total of 40 multidisciplinary HCPs participated in the study. Focus group participants were predominantly female (86%), aged 29–57, working from a public hospital within a specialist severe asthma clinic in New South Wales (NSW). There were equal numbers of male and female interviewed, they were aged 31–67, mostly working in a public hospital, with a range of 5–20 years of clinical experience. Participants represented six health professional groups, mostly from the respiratory medicine and nursing.

### 3.2 Inductive and deductive themes

**Fig 2:**
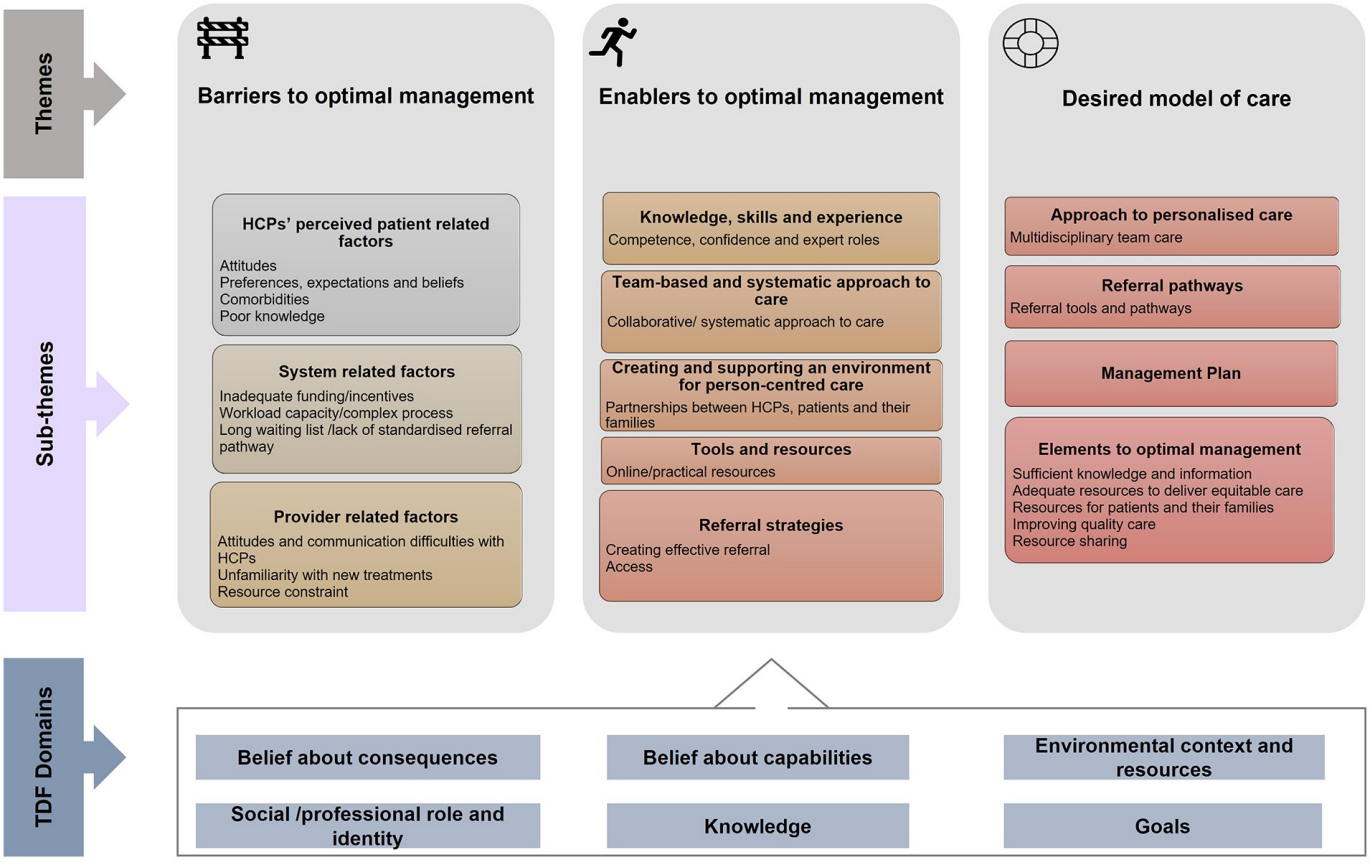
Emerging themes and sub-themes identified, and predominant TDF domains. HCP = healthcare professional. TDF = Theoretical Domains Framework.

We identified three major themes, (i) Barriers to optimal management, (ii) Enablers to optimal management and (iii) Desired Model of Care, and 12 sub-themes (Fig 2).

The TDF mapping identified six predominant TDF domains: (1) belief about consequences; (2) environmental context and resources; (3) belief about capabilities; (4) social/professional role and identity; (5) goals; and (6) knowledge. Several less dominant TDF domains were identified within themes and subthemes. These less dominant yet nonetheless important TDF domains are described below. All TDF domains are underlined, and subthemes are shown in *italics*.

Descriptive accounts of all themes and dominant and less dominant TDF domains are summarised in Tables 3 and 4. Additional illustrative quotations are contained in the online supplement (S3 Table in S1 File).

**Table 3:** Illustrative quotations regarding barriers to optimal management in severe asthma.

Themes/sub-themes	Assigned TDF domains	HCPs^a^	Illustrative quotations
**1. HCPs’ perceived patient-related factors**			
Attitudes	Belief about consequences	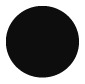	*… a lot of patients we see poorly adhere to their medication treatments so*, *yeah*, *exacerbate and end up in hospital*. (Nurse, FG)
			*significant numbers of patient who have asthma exacerbations is majority of them have very poor compliance … they don’t tend to take their medication* (oral/inhaled corticosteroids). *That’s why conditions tend to worsen … the habit as well of smoking*. (GP, P#7)
Preferences, expectations and beliefs	Belief about consequences	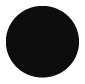	*I think it’s just resistance*. *Most of them think that they have been feeling like this for the while and it’s fine*. *They don’t recognise it as a main problem or issues*. *They think that just to get the reliever is more than good enough and that’s it*. (GP, P#7)
			*So*, *I think sometimes accepting what we say and adhering to what we say is perhaps the biggest challenge that I see in these patients*. *So*, *it’s purely because of their expectations that they think that a tablet or a puffer or injection could help them*. (RS, P#27)
	Belief about regulation	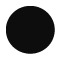	*From a speech pathology point of view*, *I cannot tell you how many people have asked me for a magic pill … I think that’s the biggest issue*. *People want something that’s a quick fix*. *They want a medication to fix something that isn’t fixable with a medication*. (SP, P#25)
			*I think there are also a group of people in whom they have asthma*, *but there’s a lot of other things going on*. *I’m particularly thinking about anxiety*, *depression*, *dysfunctional breathing*, *vocal cord problems and helping them to realise that it’s not just their asthma that’s causing a lot of their symptoms*. *I think that’s quite challenging because often people have been—have become quite fixed in their ideas*. *That’s not always their fault; often that’s because of the doctor that’s previously been looking after them that attributes every problem that they have to their asthma*. *Therefore*, *takes the approach that if they’re feeling worse than the way to deal with that is to take more asthma medications*. (RS, P#23)
	Goals	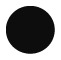	*From the outset*, *I say to them*, *‘we’re going to take on this path*, *I need you to be committed and that means you need to have your medications regularly*, *this is how we’ll stay in contact et cetera’*. *So*, *they need to have that commitment*. (RS, P#33)
			*Being able to tease out some of the damage caused by poor health and the loss of trust in your own body would be really important for us and would probably make us more effective and give some background into why we don’t get a good therapeutic relationship with some patients*. *Unfortunately*, *they don’t come back*. *They don’t believe us*. (Nurse, P#2)
	Intentions	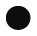	*People’s behaviour*, *I think*, *limits their access to service … they don’t engage with services*. *I think the services are there*, *but it’s how people choose to access or engage with those services*, *for various reasons*. (Nurse, P#19)
Poor knowledge	Knowledge	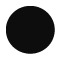	*Some are not intelligent enough to keep track of their own schedules*, *especially when you’re giving them the monoclonal antibodies*. (RS, P#28)
			*The biggest problem to lead to the asthma attack is lack of education and negligence by the customer themselves*. *They don’t understand the importance of their medication and the importance of compliance and the trigger factors*. (Pharmacist, P#3)
**2. System-related factors**			
Inadequate funding and incentives	Environmental context	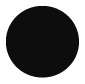	*we are scrambling for space*, *really*. *That affects waiting lists*. *so yeah*, *capacity and additional resources in terms of lung function testing … parking is a huge problem at our hospital*. (RS, P#14)
			*resource funding in the sense of my own time as a specialist*. *My hours are capped*, *and I get stretched to do other general respiratory stuff or general call*. (RS, P#33)
			*we don’t have access to electronic health records* (ED physician, P#16)
			*the biggest problem with my current workplace is the lack of bed space*. (ED physician, P#10)
Workload capacity and complex process	Environment context and resources	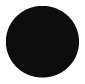	*That would be some of the paperwork and bureaucratic obstructions for getting biologics*. (RS, P#28)
			*All the things I arrange for biological therapy are out of my time and my resources and take up time of GPs and GP nurses as well*, *which they’re not very well reimbursed for*. (Nurse, P#2)
Long waiting list and lack of standardised referral pathway	Environment context and resources	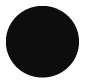	*One is it often takes a long time for patients to get into the clinic; there’s quite a long waiting list*. (RS, P#23)
			*There is no direct pathway for us in the emergency department to refer to a respiratory physician*. (ED Physician, P#8)
			*A lot of the referrals come quite late; that there’s a lot of people who struggle in the community with asthma for quite a long time before they get picked up in hospital or—is kind of a last resort if the GP is struggling*. (RS, advanced trainee, FG)
	Belief about consequences	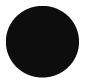	*So*, *what I’m always fighting against is why is it someone with—not that I don’t think cancer is important—but why is it that someone thinks someone with cancer—with early cancer or advanced cancer—with very little treatment options—why is it that they need to be seen within 2 weeks and yet one of our most severe asthmatics who has also has burdened symptoms can wait a year to be seen when there’s a lot more that I can do for them*. *Get them back to work*, *for example*. (RS, P#33)
			*we have no clear pathway at my hospital for psychology input into management of patients with severe asthma*. *We really are dependent on fairly non-specific clinics*. (RS, P#14)
			*Probably*, [issues]*—it would be—the hospital I worked in*, *there was really no clear pathway or direct obesity management procedure*. *It can make you feel incompetent at times*. (Nurse, P#11)
**3. Provider-related factors**			
Attitudes and communication difficulties between HCPs	Belief about consequences	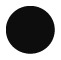	*Sometimes the barrier is*, *it’s hard to talk to consultants because they are* [snobs] *… it’s hard to approach them*. (Nurse, P#18)
			*most of those physicians doesn’t* [sic] *want to have a telehealth conference for these patients*. *We have only very few specialists who accept a telehealth consultation*. *None of them is a chest physician*. (GP, P#7)
			*telehealth for sick patients is not good*, *and I’m not a believer in telehealth for new patients*. *So*, *in private*, *I’m not doing any telehealth for new patients because it’s quite superficial and you miss things*. (RS, P#30)
	Skills	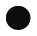	*I’d probably say some counselling and cognitive behavioural therapy training*. (Nurse, P#2)
Unfamiliarity with new therapies	Knowledge	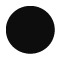	*Well*, *with biological therapies*, *I want to be honest with you*, *I’m not fully knowledgeable with that*. (GP, P#31)
			*One big area of need is in the emergency department*. *They’re often managed by interns who have very little knowledge about asthma itself let alone severe asthma*. (RS, P#14)
	Belief about consequences	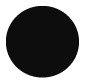	*The second problem with emergency departments is that they really promote overuse of salbutamol*. *So*, *patients leaving the emergency department*, *in my hospital*, *as in most other hospitals around Australia*, *are given a short-term action plan that calls for absolutely massive doses of short-acting beta agonist*. *It’s partly driven by the quality standards that relate to four hours*. (RS, P#14)
			*The only thing I’d like to share is that I think having worked in the emergency department in that setting for 5 years—that means that we—there is a big issue around overtreating things*. *That is absolutely—definitely one of those things*. (Nurse, P#21)
Resource constraint	Belief about consequences	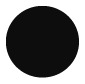	*I think probably the greatest limitation is when you have multiple problems*. *You need to try and refer people or get access to other services*. (RS, P#26)
			*there’s a lot of research on the treatable traits model*, *which we know is excellent*, *but it is—I think part of the basis of all this research is*, *how do you implement that in a time limited clinic*? *I honestly think that’s really hard*. *I don’t think that there’s a standardised way that every physician goes through treatable traits*. (RS, advanced trainee, FG)
	Environmental context and resources	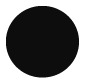	*Our clinic is very crowded and sometimes the junior doctors who help out in the clinic find that a little bit tricky trying to squeeze everybody in and all that sort of thing*. *But another one is*, *I’m the only physician in the severe asthma clinic*. *So*, *if I’m away—if I’m unwell or if I’m away on holidays or at a conference or something*, *the clinic gets cancelled*, *basically*. (RS, P#23)

*Note*. TDF = Theoretical Domains Framework; HCP = healthcare professional; P# = participant no.; FG = focus group; GP = general practitioner; RS = respiratory specialist; SP = speech pathologist; ED = emergency department.

^a^ From 40 HCPs.

Number of respondents who talked about a sub-theme: 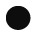 1–5, 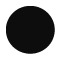 6–10, 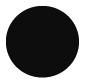 >10.

**Table 4:** Illustrative quotations regarding enablers for optimal management.

Themes/sub-themes	Assigned TDF domains	HCPs^a^	Illustrative quotations
**1. Knowledge, skills and experience**			
Competence, confidence and expert roles	Knowledge	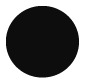	*I think my strength is that I try and minimise all the contributory factors of the comorbidities*, *which I think is quite a big strength of me because I’m used to seeing patients with comorbid conditions*. (RS, P#27)
			*I suppose my biggest strength is a very good knowledge in medicine practice in terms of severe asthma*. (ED specialist, P#16)
	Skills	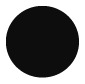	*So*, *I know how to assist them like doing the deep breathing*, *giving a nebuliser and salbutamol and then also how to get a quick response or quick review from different doctors* [sic] *…* (Nurse, P#18)
	Social and professional identity	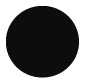	*I’m an emergency physician*, *so my role is to treat patients who come in with acute and severe asthma*. *The acute exacerbation is really our bread and butter*, *so we assess them when they come into the emergency department*, *initiate treatment and make a plan with them based on whether they’re severe enough to need intensive care on the ward*, *or we have a quick turnaround and are able to stretch them and get them home*. (ED specialist, P#10)
			*I run a severe asthma clinic … that involves assessment of people who are thought to possibly have difficult to manage or severe asthma*. *Then assessment of their various comorbidities and the issues that have been contributing to their symptoms*. *Then considering appropriate treatments for them*. (RS, P#23)
			*I refer to specialists* [otolaryngologist or respiratory physician] *and the reason why we do that is because we’re working at the level of vocal cords*. *We need to make sure there’s no laryngeal pathology before we start working with someone because we might be masking something else that’s a little bit sinister there*. (SP, P#25)
			*… as a primary care physician*, *so I’m doing mostly all of the management of asthma*, *with regards to prevention and treatment*. *I usually refer to a respiratory specialist when I know that the patient has difficulty controlling their asthma*, *despite being compliant on the medications and that the frequencies become recurring*. (GP, P#31)
			*I think having the ability to combine research with clinical practice is probably the biggest strength*. (RS, P#26)
	Belief about capabilities	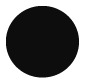	*I’m able to assimilate new information rapidly and instigate or translate whatever the best practice and treatments are into the service we currently are able to provide and within the borders of what we can do here in our metropolitan centre severe asthma clinic*. (Nurse, P#3)
**2. Team-based and systematic approach to care**			
Collaborative and systematic approach to care	Belief about capabilities	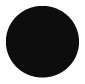	*I think the MDT approach to make sure that you are diagnosing and managing the other confounders*. (RS, P#33)
			*Having a team-based approach is really important*. *So*, *having the staff that we have in the emergency department*, *who are also familiar with the protocols*, *is fantastic*, *because we’re all on the same page right from the very beginning*. (ED specialist, P#10)
	Belief about consequences	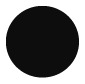	*Team-based approach is the norm for my practice … Different members of the team doing different jobs*, *I think that’s definitely the most effective*. (ED specialist, P#10)
			*It’s not just nurses working separately*, *allied health separately*. *Our physicians really much value their team who they work with and realise how important it is to have absolutely everyone on that team*. *So*, *I think we already have that magic sort of collaboration there*, *and I guess magic wand of continued collaboration*, *I think is important*. (SP, P#25)
	Reinforcement	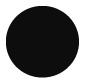	*Being able to have at the back of mind a team-based approach in the resuscitation area and our nursing staff who are also experts at helping manage our severe asthma*. (ED specialist, P#13)
	Goals	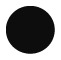	*It is trying to have a systematic approach in terms of assessing severe asthma*, *trying to characterise critical characteristics of* [a phenotype] *and then trying to direct them to optimise the management based on those clinical manifestations and then direct them to the most appropriate therapy*. (RS, P#32)
**3. Creating and supporting an environment for person-centred care**			
Partnerships between HCPs, patients and their families	Belief about capabilities	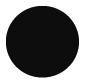	*I think being able to consider all the different possible options that are contributing to people’s symptom burden*. *Trying to be careful*, *thorough*, *trying to take a patient-centred approach with everything that we do*. *Trying to advocate on behalf of the patients and get the best possible outcomes for them*. (RS, P#23)
			*I ask patients*, *before I assess them*: *if we could do anything for your respiratory disease or respiratory issues*, *what would it be*, *in a sentence*. *That’s something that I keep at the front of the assessment*. (Nurse, P#2)
	Intentions	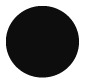	*I think one of the most effective things that any health professional can do—and I do this—just listening to them and validating what they say*. (Nurse, P#22)
			*I’ve certainly had a lot of discussions and a lot of involvement with patient’s carers and family members*, *both with these family members and carers wanting to advocate for the patient*, *with more questions and better understanding or just being a strong support*. *But also engaging the carers to understand the importance of the treatment*. (Nurse, P#2)
			*… so we involve carers in the education process* [inhaler technique, written asthma action plan]. (RS, P#33)
	Goals	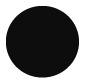	*Carers are often worried*: *they’re aware that the person they’re in with has had previous severe asthma exacerbations*, *it’s quite frightening for them*. *It’s a stressful experience*, *scary experience for them*. (ED, P#16)
			*I guess we haven’t really—one thing we probably haven’t addressed as well as we might would be mental health needs of the carers*. (RS, P#23)
**4. Tools and resources**			
Online and practical resources	Environmental context and resources	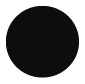	*I think I just would reiterate that the Severe Asthma Toolkit and other efforts like that have been very important*. *We’re really trying to apply all those as best as we can to our practice*. (RS, P#15)
			*I’m a big fan of the online tools—the Severe Asthma Toolbox and things*. (RS, P#30)
			*Yes*, *the GINA guidelines are very important—or the GINA strategy—has a lot of information there including severe asthma*. *I think their most recent algorithms are very helpful that have clinical pathways which are useful globally*, *need to adapt that locally*. (RS, P#15)
	Belief about consequences	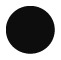	*I think a model that allows greater access and that can involve telehealth*, *which is why we’ve started doing that here at the university in our voice clinics*, *so far locations can actually access the same services that people in the cities can … our speech clinic has these amazing Zoom facilities within that clinic*. (SP, P#25)
**5. Referral strategies**			
Creating effective referral	Intentions	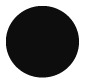	*So*, *it’s trying to work out which are the highest priorities of the treatable traits that need to be looked at*. *The most symptomatic and the most severe*, *and then we would make those referrals*. (RS, P#15)
	Memory, attention and decision-making	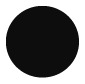	*Depending on their other problems*. *I think I would refer*. *But sometimes*, *if the magnitude of the problem is not that much*, *you want to concentrate on what you are doing first before you refer it on*. *So*, *it depends on the other comorbid problems and their magnitude*. (RS, P#27)
	Social and professional role and identity	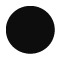	*we trust our respiratory physicians and they trust that we are referring somebody appropriately to them*. (SP, P#25)
Access	Environment context and resources	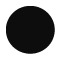	*I refer depending on how I know I can access services*. *For speech pathology*, *it’s definitely really quick and efficient and good using the public referral*. (RS, P#30)

*Note*. TDF = Theoretical Domains Framework; HCP = healthcare professional; RS = respiratory specialist; P# = participant no.; ED = emergency department; SP = speech pathologist; MDT = multidisciplinary team; GP = general practitioner; GINA = Global Initiative for Asthma.

^a^ From 40 HCPs.

Number of respondents who talked about a sub-theme: 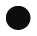 1–5, 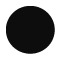 6–10, 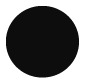 >10.

## 4 Theme 1 barriers to optimal management

Barriers to personalised care included three sub-themes and refers to the complex nonlinear interactions that occur between what HCPs perceived as patient-, system-and provider- related factors.

### 4.1 Barriers to optimal management

#### 4.1.1 HCPs perceived patient related factor

Within this sub-theme, the dominant TDF domains was ‘belief about consequences’ ‘behavioural regulation’, **‘**knowledge’ and ‘goals’, respectively (Table 3). Clinicians highlighted issues they ‘blamed’ the patient for, such as non-adherence, and a lack of self-activation in their disease management.

*4*.*1*.*1*.*1*. *TDF domain*: *Belief about consequences*. HCPs attributed poor outcomes to patients’ attitudes, expectations and beliefs about their treatment and comorbidities. These factors were seen as barriers as they impact the patients’ ability to self-manage their care or treatment regimen. Medication non-adherence, and lifestyle factors were perceived as the biggest threats, leading to substantial worsening of disease and frequent emergency department (ED) presentations.

*the biggest problem is the non-compliance of patients*, *with regards to their medications* (oral and inhaled corticosteroids).

(GP, P31)

The perception of some HCPs was the patients did not ‘take ownership’ when prescriptions were due, or they wanted a ‘quick fix’ therapy; this was a source of frustration for some clinicians.

*So, it’s a little bit frustrating that patients, not all patients, but a lot of patients aren’t taking that responsibility to—or they don’t have insight to when their scripts run out, so that’s a very—for me personally, that’s a bit frustrating, because you want to be the best you can be for the patient, provide the best care [sic]*.

(*Nurse, P4*)

and

*I think my barriers at times is that the patients, they’re expecting a treatment, as in a tablet or an injection [sic]. Rather than being able to go back and change their lifestyles*.

(*Respiratory specialist, P27*)

HCPs raised a growing concern about comorbidities (i.e., obesity, dysfunctional breathing, anxiety and depression) as a rising challenge, increasing the complexity of asthma management. An additional belief within this TDF domain, from the HCPs’ perspective, was the urgent need to address obesity-related comorbidities. The importance of multidimensional assessment and timely access to allied health services to address such comorbidities was also recognised.

*4*.*1*.*1*.*2*. *TDF domain*: *Behavioural regulation*. Clinicians discussed how they thought patients held a ‘fixed mindset’ about their disease. This was viewed as a challenge when considering the presence of comorbidities that confound and complicate asthma. Several clinicians believed that patients should not be blamed for their treatment failures but rather supported in a patient-centred systematic manner.

*4*.*1*.*1*.*3*. *TDF domain*: *Goals*. HCPs believed that patient-centred goal-setting was essential for ‘encouraging’ patients to engage and remain committed in their treatment plans. Unfortunately, several HCPs felt that a disconnect in patient-clinician therapeutic relationships existed despite their efforts.

*4*.*1*.*1*.*4*. *TDF domain*: *Knowledge*. HCPs perceived that patients lacking knowledge about their condition was a barrier to optimal outcomes being achieved.

*… lack of understanding and culture or the behaviour as well of the patients*.

(GP,P7)

*4*.*1*.*1*.*5*. *TDF domain*: *Intentions*. HCPs viewed patients’ unwillingness to engage with medical services as another barrier.

#### 4.1.2 System related factors

Central to this sub-theme is the ‘environmental context and resources**’** domain (Table 3). The TDF domain ‘belief about consequences**’** was also identified. Clinicians discussed how they saw healthcare systems as a barrier to providing optimal severe asthma care. Clinicians discussed the ways healthcare systems were a barrier to providing optimal severe asthma care.

*4*.*1*.*2*.*1*. *TDF domain*: *Environmental context and resources*. HCPs consistently described a system ‘plagued’ by long waiting times, inadequate resources, and delays in care.

*It’s lack of resources … there’s limited access to space and to additional team members and so on. That’s our biggest problem*.

(Respiratory *s*pecialist, P15)

Many reported inadequate staffing and resources as a barrier, including access to services (pulmonary function test, links to electronic health records and, inaccessible parking areas). There was a concern regarding excessive workload or increased paperwork requirements for ‘bureaucratic’ processes associated with biologic therapies, increasing HCPs’ occupational stress or burnout.

*4*.*1*.*2*.*2*. *TDF domain*: *Belief about consequences*. Some respiratory specialists felt frustrated when they were viewed as a ‘last resort’. This related to their perception that the GP referred too late. Referral delays were viewed as a barrier causing dissatisfaction among respiratory specialists and preventing review in a timely manner. This feeling of dissatisfaction was shared among emergency specialists, due to limited referral pathways for rapid access. Other HCPs believed that to facilitate patient uptake and engagement in services, efforts to reduce waiting lists were necessary. Another ‘belief about consequences’ pertained to the absence of standardised asthma guidelines, specifically for assessing and managing obesity-related comorbidities. This left some HCPs feeling ‘incompetent’.

Some HCPs indicated that the absence of key performance measures for severe asthma was a driver of patients not being able to access care in a timely manner.

#### 4.1.3 Provider-related factors

The final sub-theme within the barriers theme, related to the clinicians themselves, particularly concerning communication between services and training. Within this sub-theme the TDF domains of ‘environmental context and resources,’ ‘belief about consequences,’ ‘knowledge’ and ‘skills’ were identified.

*4*.*1*.*3*.*1*. *TDF domain*: *Belief about consequences*. Poor communication between clinicians was proposed as hindering MDT functioning and positive patient outcomes. Some rural GPs reported difficulties streamlining referral processes when respiratory specialists did not accept telehealth consultations. Rural GPs perceived that this is a barrier to effective referral and optimal care. However, a few specialists expressed concern that some aspects of care could only be attended face-to face, such as physical examination for very sick and new patients. Several HCPs expressed reluctance collaborating with other clinicians who have been identified as having difficult personality traits.

*There’s a particular physician locally who can be quite difficult to speak to, and I think people are reluctant to call when there’s a personality type at the end of the phone that would make people reluctant*.

(ED *s*pecialist, P8)

Additionally other HCPs reported a concern that some GPs were unwilling to refer older asthma patients to specialist care.

*Some GPs, particularly with older clients, are not even happy about sending them off to a specialist, because they think, ‘well they’re 80 or 90 so, you know? I’ll handle it. I’ll handle it’. Once again, it’s people not being listened to*.

(Nurse, P22*)*

A great concern perceived by some respiratory specialists was the over ‘promotion’ of short-acting beta-agonist (SABA) therapy in the ED. These specialists perceived that this was partly driven by the’ 4-hour rule’ to either admit, transfer or discharge patients within the ED. This performance indicator was perceived to have contributed excessive pressure on the ED staff, resulting in the delivery of maximal therapy to patients regardless of their need, which was believed to have contributed to SABA overuse.

*4*.*1*.*3*.*2*. *TDF domain*: *Environmental context and resources*. Whilst many HCPs believed in the benefits of applying a ‘treatable traits’ approach, they felt that this approach failed to address the main barriers of time constraints. Limited and highly variable access to allied health across settings remained a significant issue. Many reported that fluctuating staff levels or reduced staffing was an ongoing barrier and a struggle for HCPs to deliver best-practice care.

*4*.*1*.*3*.*3*. *TDF domain*: *Knowledge and skills*. Whilst knowledge and skills are determinants for effective and efficient MDTs, some HCPs reported a lack of knowledge relating to new asthma treatments and approaches to care. For example, some HCPs highlighted their desire for increased training in counselling and cognitive behavioural therapy.

## 5. Theme 2: Enablers to optimal management

Within the enablers theme, there were five sub-themes. Between the barriers to and enablers for optimal management themes, there was an overlap of identified TDF domains. Participant quotes for these sub-themes are presented in Table 4.

### 5.1 Knowledge, skills and experience

#### 5.1.1. TDF domain: Knowledge and skills

HCPs described individual clinical expertise and the discipline-specific **‘**knowledge’ and ‘skills’ as facilitators to providing the highest standard of care.

#### 5.1.2. TDF domain: Belief about capabilities

HCPs reported awareness of their strengths and competences when treating patients during acute attacks. HCPs noted that their ability to ascertain patient needs and understand the evidence supporting treatments empowered them to promote optimal care.

#### 5.1.3. TDF domain: Social professional role and identity

HCPs acknowledged their expertise, role, and scope of practice in determining their function in severe asthma care. For example, respiratory specialists strongly emphasised that their role included confirming and assessing severe asthma, identifying comorbidities, and prescribing pharmacotherapy including biological therapies, whilst ED specialists highlighted their role in managing acute asthma attacks. GP and other allied HCPs reported referring patients to respiratory specialists. Allied HCPs saw that they had a designated role in working with patients to improve asthma control with self-management education, and in addressing comorbidities (e.g., laryngeal pathology). Each group expressed a strong disciplinary role identity and was supportive of each other’s professional skills and contribution. These disciplinary roles and their unique clinical expertise drove HCPs’ motivation to provide the best practice and maintain clinical excellence. Some HCPs also described their clinical research roles as enabling to drive improvements in evidence-based care.

### 5.2 Team-based and systematic approach to care

#### 5.2.1. TDF domain: Belief about capabilities

The support and active involvement of multidisciplinary HCPs in severe asthma management was an important enabler. Most HCPs believed that working in collaborative teams reduced inefficiencies and errors.

#### 5.2.2. TDF domain: Belief about consequences

Team engagement brought together HCPs from diverse disciplines to deliver personalised and holistic care. This multidisciplinary approach was perceived to counteract any ‘silo effect’; that is working in isolation.

#### 5.2.3. TDF domain: Reinforcement

Collaborative and systematic care resulted in a positive experience for most HCPs, generating a synergistic influence of collective knowledge and skills. This was perceived as a critical enabler in improving patient outcomes.

#### 5.2.4. TDF domain: Goals

HCPs wanted the best outcome for their patients, highlighting their intention to provide systematic approaches to care; this was considered as one of the most important enablers to ensure correct diagnosis and identification of treatable traits in severe asthma.

### 5.3 Creating and supporting an environment for person -centred care

#### 5.3.1. TDF domain: Belief about capabilities

HCPs recounted their experience of involving patients and their families in treatment and care planning. Person-centred care was encouraged within clinical practice through positive role modelling and respecting patient values and preferences. The increase in HCPs’ confidence in communicating with patients, active listening and empathy was highlighted and are believed to be effective approaches to managing patients.

HCPs reported numerous encounters with carers wanting to advocate for their loved ones. Whilst there was no doubt that support roles are valuable, a few HCPs found some carers ‘difficult to gauge’ and other carers questioned treatment efficacy, and some thought their care recipients were exaggerating their symptoms.

*But there’ve been times when I’ve thought that the carers have not been helpful*. *They’re usually times when the carers have been disbelieving of what their* [care recipients actually feel and doubts whether just exaggerating].

(Nurse, P22)

HCPs believed that engaging carers when devising a care plan would likely lead to the best outcome for the person they care for.

#### 5.3.2. TDF domain: Intentions

HCPs highlighted their intention to offer education sessions and training to build the knowledge and skills that carers need to support optimum care.

#### 5.3.3. TDF domain: Goals

HCPs raised awareness of carers’ well-being needs, particularly when witnessing acute severe attacks. Meeting the mental health needs of carers was a goal for many HCPs, but they reported gaps in the system for carer support.

*So*, *we involve carers in the education*, *but*, *unfortunately*, *we don’t have a pathway*, *for example*, *where we go* [if] *this carer is under a lot of stress*, *can we organise a clinical psychologist review*? *We don’t have that*. *Unless they have a system with us*, *we don’t do that*.

(Respiratory *s*pecialist, P33)

### 5.4 Tools and resources

#### 5.4.1. TDF domain: Environmental context

HCPs identified a range of clinical resources they used in their practice. For example, respiratory physicians seemed to widely access the severe asthma toolkit [33]. Other professions (GPs, ED specialist, Nurses and Allied HCPs) used online tools like (National Asthma Council, Asthma Australia, Global Initiative for Asthma, Therapeutic Guidelines and The Royal Children Hospital (RCH) -Clinical Practice Guidelines). These HCPs reported that such resources boosted their clinical competence and enhanced clinical decision-making.

#### 5.4.2. TDF domain: Belief about consequences

Most HCPs saw an opportunity to embed telehealth into their practice, thereby promoting timely access to healthcare, particularly in rural and remote regions. Delivering healthcare via telehealth videoconferencing was perceived by some HCPs as enabling and extending day-to-day clinical practice whilst allowing simultaneous, timely and efficient collaboration with local healthcare teams.

### 5.5 Referral strategies

#### 5.5.1. TDF domain: Intentions

Respiratory specialists who regularly referred to allied health networks (physiotherapist and speech pathologist) noted their intent to use the bespoke approach to refer as often as possible. Respiratory specialists believed that dealing with the most troublesome and burdensome treatable traits is an effective strategy for promoting a systematised process.

#### 5.5.2. TDF domain: Memory, attention and decision-making

HCPs noted factors that influenced their decision to refer. GPs referred to specialist services specifically when a patient is unable to attain asthma control and when comorbidities complicate their asthma. Some factors that lead to a lack or delay of referral related to clinical judgement, assessment and the magnitude of comorbid problems.

#### 5.5.3. TDF domain: Social/Professional role and identity

HCPs believed that a timely referral was a critical component of their role. A relationship of trust and confidence amongst HCPs influenced referral processes. Referring HCPs indicated that having an awareness of how they can access services ultimately influenced their referral choices.

## 6 Theme 3: Desired model of care

Clinicians discussed a desired model of care that reflected MDT-based proactive care, supported by adequate resources, effective systems, enabling technology, and a culture of person-centred care, irrespective of geographical remoteness to better meet the health care needs of people with severe asthma. Participant quotes relating to this are presented in Table 5 and online supplement (S4 Table in S1 File).

**Table 5:** Illustrative quotations regarding the desired model of care in severe asthma.

Theme/sub-theme	Illustrative quotations
**Approach to personalised care**	
Multidisciplinary team care	*So*, *if I had a magic wand in my hand*, *I would want a multidisciplinary team available in the clinic*. (RS, P#23)
	*it’s important for the GP to be involved because they’re the ones that are going to be looking after the child in the community and long-term*. (Nurse, P#6)
	*It’s all very nice to have a severe asthma clinic and a model of care*, *but the reality is that*, *as soon as something happen that’s considered a non-necessary service … I think that you need that mixture extra of private and public because the reality is that there are big accessibility problems in public*. (RS, P#30)
**Referral pathways**	
Referral tools and pathways	*Maybe as part of the referral thing*, *like*, *discrete criteria with that*. (Physiotherapist, FG)
	*to have more proforma templates on the referral process*. (RS, P#32)
	*The basics … the assessment before they come to the severe asthma clinic*. *As in*, *like nurse assessment*, *FeNO and PFTs*, *exacerbation history*, *OCS use and all that before they get to the SAC meeting*, *because then it’s more helpful for everyone*. (Nurse, FG)
**Management plan**	*So*, *we’ve assessed them*. *Yeah*, *so that’s happened in MDT and then we all coordinate all those multidisciplinary things*, *then starting treatment*, *depending what the outcome of that assessment is*. *Then regular follow-ups depending on the outcomes*. *I guess that needs to be communicated back to the GP*. (Nurse, FG)
	*if I had a magic wand*, *I would like a pre-screening process*, *like the one developed in Melbourne*. *And I would like a joint case discussion meeting*. (RS, P#14)
**Elements to optimal management**	
Sufficient knowledge and information	*I want to learn more about the biological therapies and what are the options for us GPs with regards to giving those medications*. (GP, P#33)
Adequate resources to deliver equitable care	*The other thing is that the government should somehow subsidise as an add-on to access allied health services*, *because*, *if you limit only the allied health services for five times a year—and some of these allied health services even put significant gaps in there—the patient cannot avail them or able to utilise them because of the cost as well*. (GP, P#7)
	*An electronic health record which allows ED to access not just the discharge summary*, *but it would be really good to have access during their stay in the emergency department*. (ED specialist, P#16)
	*I think it’d be really lovely to see a lot more collaboration between government agencies and maybe private agencies and how we can get people seen a little bit quicker*. *So*, *it would be lovely to see if there was a little bit more of a link and ease of access of information* [with the electronic health record]. (SP, P#25)
	*Oh*, *well*, *something really simple would be a list of all of the local health districts in New South Wales and the contact names*, *numbers*, *fax numbers for the relevant departments*. (RS, P#14)
Resources for patients and their families	*I think if there were resources like little video clips*, *to show patients how to use puffers*, *like little YouTube videos or something like that*. *Where we teach them*, *and sometimes we may not have access to a respiratory liaison nurse who could teach them that*. (RS, P#27)
	*Well if we had a chart*, *we can highlight for the patient the importance of their medication then that would be easy … Another good option would be an app on the phone for example*. *That would be very clever*. (Pharmacist, P#3)
	*Maybe visual and audio material is going to be more effective rather than paper-based material*. (RS, P#27)
Improving quality care	*If we were looking more at a patient-centred approach and patients who have known severe asthma*, *so have current asthma that has acute attacks frequently*, *to actually have something with them or on their person*, *that had a card* [identification reference/passport] *that you can quickly look at*. *It’s just something that says*, *‘I can’t speak for myself right now because I’m sick*, *but this me*, *you don’t have to go through* [my electronic medical record] *for the next 20 minutes’*. (ED specialist, P#13)
	*I think one of the things we fight against—there is no key performance index for asthma or airways disease*. (RS, P#33)
	*Yeah*, *if we had pulmonary rehab for our patients because we send half our patients or the majority of our patients home with asthma or COPD—if we could get them linked into pulmonary rehab*, *I know there is benefit in that*, *and I don’t think we have that right now*. (ED specialist, P#9)
Resource-sharing	*I think in terms of being able to have updates from centres that are doing it more regularly or compare how we do it in different centres with different resources in terms of how we can improve our overall care*, *that would be good*. *Places that don’t have a severe asthma service but want to set one up*, *then being able to provide training and hints about how you can set it up would be one way*, *as sort of a webinar or sort of training workshop*. (RS, P#32)

*Note*. RS = respiratory specialist; GP = general practitioner; P# = participant no.; FG = focus group; SAC = severe asthma clinic;

FeNO = fractional exhaled nitric oxide; PFT = pulmonary function test; OCS = oral corticosteroid; HCP = healthcare professional;

MDT = multidisciplinary team; ED = emergency department; SP = speech pathologist;

COPD = chronic obstructive pulmonary disease.

**Fig 3:**
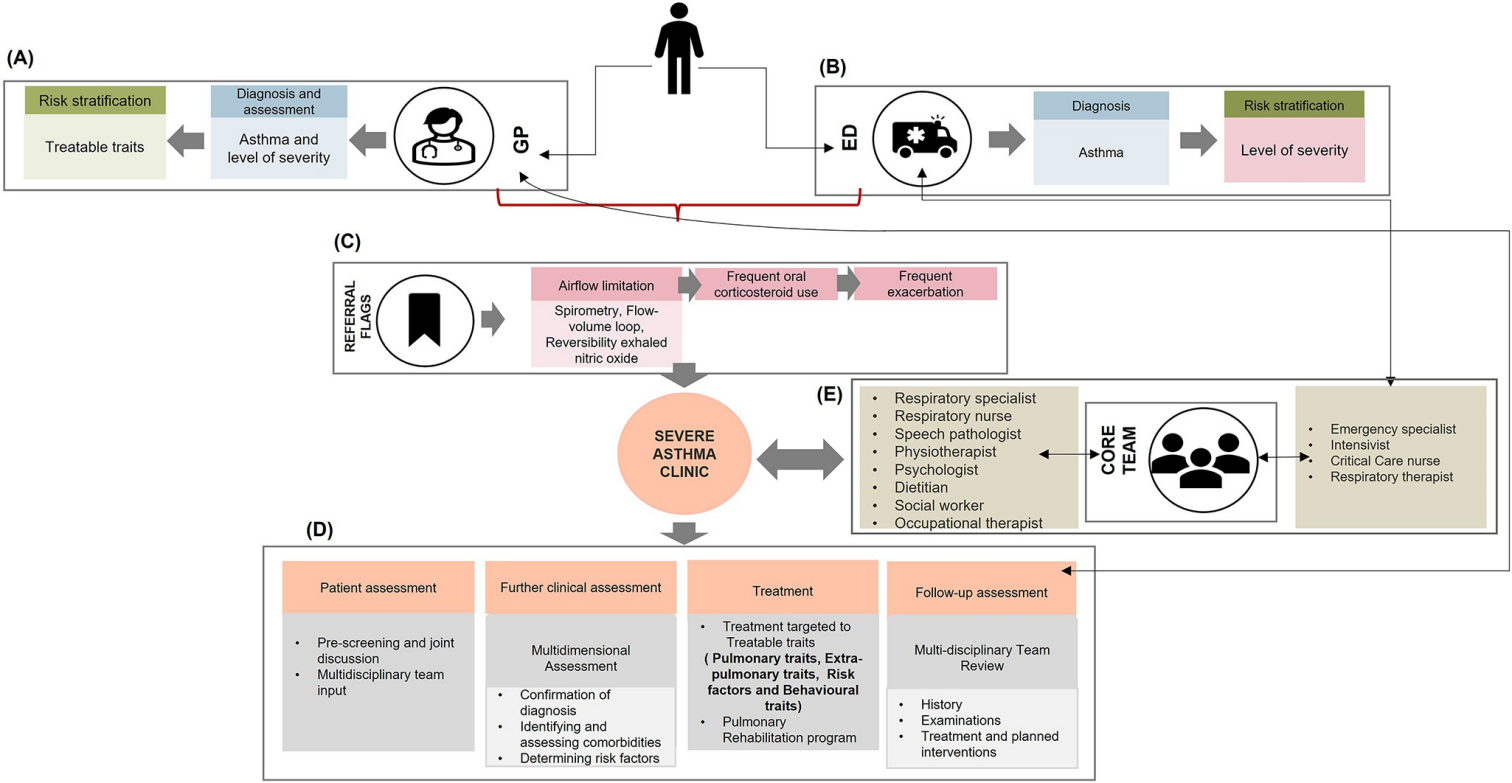
HCPs’ proposed care pathways in severe asthma. (A) GP pathway, (B) ED pathway, (C) Referral criteria to specialist care, (D) Multidimensional assessment and individualised management, (E) MDT involved in severe asthma care. GP = general practitioner; ED = emergency department; MDT = multidisciplinary team.

Based on the HCPs’ responses, a conceptual care pathway in severe asthma has been proposed (Fig 3).

The Desired Model of Care theme included the following sub-themes:

### 6.1 Approach to personalised care

Respiratory specialists proposed a standardised MDT approach as a way of optimising care. Having a ‘one-stop-shop’ with nurses and key allied health (Fig 3) embedded in dedicated severe asthma centres to provide efficient care in a timely manner.

*if I had a magic wand in my hand, I would want a multidisciplinary team available in the clinic*.

(Respiratory *s*pecialist, P23)

This view was shared by other disciplines who felt that a collaborative team approach improved patient satisfaction and health outcomes. As the COVID-19 pandemic accelerated, some HCPs believed that optimising partnerships between the public and private health sectors are critical to meeting the needs of patients and mitigating short or long-term challenges in accessing health services.

### 6.2. Referral pathways

To streamline referral from both primary and tertiary care streams, it was proposed that ‘discrete criteria’ should be established to prompt GPs or ED specialists to refer to specialist care.

*Maybe having referral criteria that—for when a GP should be provoked to think to refer it to a respiratory specialist. Because I think, at the moment, a lot of it’s on their vibe of, ‘oh, I’m not managing’*.

(Advance*d t*rainee, FG)

Specialists indicated their preferences for what should be included in referrals, suggesting relevant clinical history (i.e., exacerbation history, corticosteroid use), investigation results (fractional exhaled nitric oxide (FeNO), Pulmonary Function Test), and a detailed reason for referral. Patients meeting the proposed criteria could be expedited to the severe asthma service. There was a consensus that GP education was needed regarding the treatable traits that commonly coexist in severe asthma, and that the outcomes from assessments and planned interventions should be communicated back to the referring GP. Other HCPs proposed the inclusion of pulmonary rehabilitation as part of the management plan for severe asthma.

### 6.3 Management plan

A pre-screening process and a combined MDT meeting and discussion were viewed as critical to enhancing management and treatment plans in specialist clinics. Depending on the outcomes and decisions arising from the assessment, targeted therapy can be initiated with regular follow-up thereafter that includes inhaler technique and medication adherence (Fig 3).

### 6.4 Elements to optimal management (suggested enablers)

HCPs were invited to suggest possible enablers for implementing optimal management. Table 6 summarises the five sub-themes of categorised recommendations and their alignment to the enablers theme and to the TDF domains.

**Table 6:** Suggested enablers for optimal management.

Suggested enablers	Capabilities/recommendation	Avenues	Enabler themes	TDF domain
Sufficient knowledge and information	Access to counselling and cognitive behavioural therapy training Delivery of adequate information on available treatments Simulation training	Workshops Online toolkit Community-based asthma management program Education session and simulation training Online modules	Knowledge, skills and experience	Knowledge Skills Social and professional role and identity Belief about capabilities
Adequate resources to deliver equitable care	Subsidised allied health services (safety net) Ensure clinical information is shared across systems to provide clinicians with a single access/portal to all available patient information to inform clinical decision-making Online directory for referral within a local health district	Specialist bulk-billing clinics Link to electronic health records List of local health districts	Online tools and resources	Environmental context and resources Reinforcement
Resources for patients and their families	Increase health promotion Use of technology	Pulmonary rehabilitation Pamphlets and other paper-based material Visual and audio materials	Creating and supporting an environment for patient-centred care	Belief about capabilities Intentions Goals
Improving quality care	Develop automatic coordination and tracking of patients’ identities and access cards Healthcare professionals writing appropriate referrals Key performance indicators in severe asthma	Patient identification, severe asthma (passport) card Severe asthma bracelet Proforma templates Mental checklist Electronic checklist Pulmonary rehabilitation	Team-based approach Referral strategies	Belief about capabilities Goals Social and professional role and identity Memory, attention, and decision process Intentions
Resource-sharing	Collaborative interdepartmental meetings Severe asthma clinics to share resources to promote efficiency	Online workshops Joint case discussions Interdepartmental meetings	Online tools and resources	Environmental context and resources Reinforcement

A severe asthma identification card (passport) and bracelet were believed to be important in improving care needs by providing HCPs timely health care information thus promoting personalised care. Resource sharing across severe asthma clinics was viewed as an important step to improve services. Knowledge gained through benchmarking different services can be used as a guiding principle to optimise care.

## 7 Discussion

Personalised care in severe asthma is both desirable and challenging (4). We sought to determine HCPs’ perceived barriers and enablers that influence the implementation of personalised care. The findings shows that barriers span multiple levels, including HCPs’ perceived patient-, system-, and provider related factors. Using the TDF as a tool to further understand these barriers and enablers we found six overarching TDF domains: (i) belief about consequences, (ii) environmental context and resources, (iii) belief about capabilities, (iv) social/professional role and identity, (v) goals and (vi) knowledge. Analysis using the TDF has generated an increased awareness of the HCPs’ perceived barriers and enablers from a severe asthma perspective and defined which barriers need to be targeted to better implementation of a personalised care approach. This study provides an important insight in understanding the main drivers to the uptake of personalised care and guides the development of effective strategies to improve practice. To our knowledge this is the first study in severe asthma to use a theory-based framework to investigate an implementation approach.

Our results demonstrate that barriers exist at multiple levels and highlight why implementation of personalised care in clinical practice may not have been achieved in all areas of asthma and severe asthma management. Although some barriers identified differed in scope, context, and strength, the majority were collectively identified by the multidisciplinary HCPs. Our results confirm and align with previous findings [34] that clinicians perceive lack of patient engagement, adherence to treatment (oral/inhaled corticosteroids), poor illness perception, beliefs, preferences, and expectations as significant barriers to optimal outcomes. This suggests that patients’ non-adherence to prescribed medication [35, 36] remains a difficult problem to resolve and continues to be a great concern for most HCPs. With the emergence of biological therapies, the complexity of treatments for asthma patients has increased even more [26]. Interestingly a similar pattern of insights was obtained from people with severe asthma themselves wherein they reported lack of awareness and self-management and poor treatment adherence as barriers to correct and timely diagnosis of severe asthma [37]. This highlights the importance of understanding the drivers of patient behaviour change [35] including trade-off preferences [38] for developing effective personalised strategies to promote medication adherence in severe asthma [35]. In a scoping review on barriers and facilitators to medication adherence, patient desired for more open communication and better information [34]. This also highlights the importance of effective patient-clinician partnership and multidisciplinary care in severe asthma to improve patient behaviours, as opposed to deferring ‘blame’ to patients [39]. Clark et al., proposed a model of clinician-patient partnership in asthma that would enhance patient perceptions and reduce health care use [40]. Ghimire et al. reported that a single home visit by a MDT improved patient adherence to medication, clinic visits, and reduced healthcare utilisation [41]. Most of our interviewees recognised the importance of collaborative work, identifying the pharmacist’s role in promoting adherence [42].

It is not surprising that system-related factors, including waitlist and appointment delays, poor service availability and access to biomarker testing emerged as themes. These barriers are consistent and recognised in many areas of chronic disease management [43–46], and we acknowledge that whilst significant, they are not easily changed.

A review on barriers to healthcare access in Australia has focused mainly on health care access in the cancer-related research area and in understanding accessibility among metropolitan and regional populations [44]. A unique barrier highlighted by HCPs in this study was the perceived absence of key performance indicators (quality of care indicators) for asthma. A key performance indicator is a key metric that describes a situation concisely, aids in tracking development and performance, and serves as a guide to support decision making [47]. Key performance indicators can assist in identifying barriers to and enablers of the uptake of clinical guidelines for asthma management [48]. Barriers to quality improvement hinder the optimal flow of personalised care [49]. This result underpins the need to develop person-centred performance measures to address the complexity of care patients with severe asthma and their families require. From a healthcare provider level, lack of funded allied health services is a major limitation impacting practice. Standards of care are used in other countries to advocate and support the resource allocation for specialist severe asthma care. In the UK, an approach to specialised commissioning severe asthma (significant resources are focused towards a clearly defined pool of rare and complicated conditions, and small numbers of centres of excellence with broad catchment regions are especially supported) has been used [50] and this lesson could be adapted to the Australian health system. To support the commissioning of highly specialised severe asthma services, a hub and network model [50] offers the opportunity to extend equitable care in Australia [51]. The hub network provides a unique potential to improve efficiency and effectiveness by strategically centralising the most sophisticated medical services at a single site and distributing basic services via secondary sites [52]. A systematic review on innovative models of primary health care in rural and remote Australia suggests that a hub and network model may be necessary for delivering a full range of primary health care services to smaller and more remote communities [51]. A well-developed hub and network model can meet patient needs whilst promoting conservation of resources, investment returns, exceptional services, and improved market uptake [52].

Barriers resulting from sub-optimal interaction with healthcare teams and limited knowledge of new therapies were commonly reported and are consistent with previous studies [53–55]. Together, the findings from this study suggest that drivers of implementation change could both arise either intrinsically or extrinsically. ‘Intrinsic factors’ may be from within HCPs’ values or social influences, whilst ‘extrinsic factors’ refer to environmental or system influences that are often beyond HCPs’ control [56]. Given the emergence of new knowledge about patient’s lived experience of severe asthma [57–60] lack of exploratory inquiry about HCPs involvement in severe asthma management, our study is unique. Our study filled an important gap in understanding healthcare professionals’ insights, necessary to make severe asthma care more person-centred.

Enabling strategies included HCPs competence and knowledge, using evidence-based online resources and recommended guidelines as support tools. These enablers align with other literature in asthma and other diseases [16, 54, 61, 62]. Our study also revealed that HCPs valued the importance of MDT in severe asthma. Therefore, if outcomes are to be optimised, all stakeholders including HCPs, patient and carers must engaged in goal setting concurrently [63, 64]., Strategies that target both patients and their families and HCPs appear more likely to achieve better outcomes than those targeting either group in isolation [65]. Creating a person-centred approach to care that involves patients’ families and their significant others, could facilitate partnerships between HCPs, and this was considered a significant enabler [66]. Participants also recognised the impact that severe asthma has on family carers particularly when dealing with sudden severe attacks, but felt they were limited in providing care pathways. A needs assessment designed for carers of people with severe asthma is warranted to ensure their critical needs are met [67].

We present the HCPs desired model of care in severe asthma. These findings expand on previous quantitative results where HCPs involved in severe asthma management report variability in their practice [24]. The results demonstrate two things: First the use of a multidimensional assessment to identify treatable traits in severe asthma is a highly desired approach to address the heterogeneity in severe disease. Additionally, an emphasis on the role of the GP in the MDT was also highlighted, as proposed in review and commentary articles [19, 68]. GPs are central providers of care, coordinators and stewards in the healthcare system [19, 69]; however, HCPs identified gaps in referral pathways between primary and tertiary care. Addressing these gaps could promote improved access, quality, and continuity of care [70]. Second, resource sharing amongst severe asthma clinics is desired amongst HCPs to learn what works differently in various locations and settings. This further highlights the need for severe asthma standards of care. A detailed proforma template on the referral process and electronic access to medical records are preferable to allow smooth transitioning of care. In conjunction, a proposed severe asthma passport or bracelet was proposed to enable patients with severe asthma to get the right therapy when they present to the ED with acute attacks, making hospital admission more streamlined. Greenberg et al have previously reported that an asthma patient passport increases patients’ confidence in their ability to communicate their needs whilst in severe distress, and assisted HCPs in delivering timely and appropriate emergency care [71]. Under certain assumptions, this can be a solution to address gaps in delivering care in an acute care setting.

HCPs envisioned that strengthening public-private partnerships (voluntary cooperative arrangements between two or more public and private sectors agreeing to collaborate for a shared goal [72] complemented with telehealth for improved and increased health service accessibility particularly in times of crisis, such as the COVID-19 pandemic would better meet patient needs regardless of location. This suggests that proactive public-private partnerships are key elements to form sustainable health strategies to deliver optimal care [66].

Our study applied a validated theoretical framework to identify barriers to and enablers for implementing personalised care in severe asthma. Data saturation was achieved with the extensive citations and rigorous methodology, thereby combining a comprehensive inductive and deductive approach to ensure consistency and validity of the data. Using both techniques allowed for naturally identified themes to be determined and allocated to pre-selected theoretically driven domains to assist in addressing the study aims. A convergence of beliefs across different disciplines highlighted the consistencies of issues and gaps in current severe asthma management. We note several limitations. Firstly, most participants were from metropolitan regions within one geographical area of Australia, limiting generalisability to other regions. Some interviews with participants were generally shorter in duration compared to other interviews conducted after clinic hours. Additionally, perspectives from other allied HCPs might have provided additional views regarding the challenges of implementing personalised care in severe asthma. It is also possible that our focus group method may have incurred social desirability bias [73]. In addition, a focus group was conducted in a single institution. Despite these limitations, we mitigated such bias by conducting individual interviews with other disciplines which allowed further explanation of the phenomena. Nevertheless, the sample size and the combination of two qualitative data collection techniques enriched our understanding of HCPs experiences, perceptions, opinions, attitudes, and motivations in severe asthma management.

We also acknowledge that TDF is recognised as being most useful for individual level change [32]. Therefore, future research is warranted to apply other frameworks and theories such as the Capability, Opportunity and Motivation- Behavioural (COM-B) theory of change model, [74] to capture relevant factors within a system that influence behaviour change. Future research according to participant individual role (clinical roles, discipline, acute or non-acute) or gender may differ for these sub-groups therefore is warranted. Additionally, our findings could be used to inform implementation studies investigating strategies to overcome the barriers to implementing personalised care in severe asthma.

## 8. Conclusion

Barriers to personalised care in severe asthma are seen as multi-levelled. The use of theory-driven approach afforded a defined understanding of barriers and enablers that impact implementation of personalised severe asthma care. Without targeting these barriers at all three levels, it will not be possible to offer, deliver and achieve personalised care.

## Supporting information

S1 File (DOCX)
