# Supplementary material for: Using a knowledge translation framework to identify health care professionals’ perceived barriers and enablers for personalised severe asthma care
Source: PLoS One. 2022 Jun 7;17(6):e0269038. doi: 10.1371/journal.pone.0269038 (PMC9173624; doi:10.1371/journal.pone.0269038)
Supplement: S1 File — (DOCX) [file pone.0269038.s001.docx]

***Using a knowledge translation framework to identify health care professionals’ perceived barriers and enablers for personalised severe asthma care***

ELEANOR C. MAJELLANO, ^1, 3^ VANESSA L. CLARK, ^1, 2,3^ REBECCA F. McLOUGHLIN, ^2,3^

PETER G. GIBSON ^1, 2,4^ AND VANESSA M. McDONALD ^1, 2, 3,4^

ONLINE SUPPLEMENT

**Author Affiliations**

^1^*National Health and Medical Research Council Centre for Research Excellence in Severe Asthma and The Priority Research Centre for Health Lungs, The University of Newcastle, Newcastle, NSW, Australia;* ^2^*National Health and Medical Research Council Centre for Research Excellence in Treatable Traits ^3^School of Nursing and Midwifery, The University of Newcastle, Newcastle, NSW, Australia*; ^4^ *Department of Respiratory and Sleep Medicine, John Hunter Hospital, Hunter Medical Research Institute, Newcastle, NSW, Australia*

**Correspondence**: Vanessa M. McDonald, Level 2 West Wing, Hunter Medical Research Institute, Locked Bag 1000, New Lambton, NSW 2305, Australia.

Email: Vanessa.McDonald@newcastle.edu.au; Fax: +61404200

Additional Files:

S1 Table. Consolidated criteria for Reporting Qualitative research (COREQ) Checklist.

| Domain and Items | Author Comment | Location in manuscript  (page no) |
| --- | --- | --- |
| Domain 1: Research team and reflexivity |  |  |
| *Personal characteristics* |  |  |
| 1. Interviewer/facilitator  Which author/s conducted the interview or focus group? | All individual interviews were conducted by EM.  Both EM & VC conducted the Focus group. | See page 5, 2.3 Data collection |
| 2. Credentials  What were the researcher’s credentials? E.g. PhD, MD | *First Author*- MPH,BS Opto  *Second Author* - PhD,B. Psychology, Hons1,  *Third Author*- PhD,B. Nutrition and Dietetics,Hons1  Fourth Authors MBBS, FRACP, PhD, FThorSoc  *Fifth Author*: PhD, FThorSoc., BNurs, DipHthScien (Nurs) | - |
| 3. Occupation  What was their occupation at the time of the study? | *First Author:* PhD Candidate  *Second Author*: Research Fellow  *Third Author:* Research Fellow  *Fourth Author:* Senior Staff Specialist in Respiratory Medicine, NHMRC Practitioner Fellow, Co-Director NHMRC Centre of Research Excellence in Severe Asthma, Professor of Medicine  *Fifth Author:* Professor of Nursing, Academic Clinician, Co-Director NHMRC Centre of Research Excellence in Severe Asthma, Co-Director Priority Research Centre for Health Lungs  All author affiliations are listed in the title page | See page 1 |
| 4. Gender  Was the researcher male or female? | EM, VC , VM, RM -female  PG- male | - |
| 5. Experience and training  What experience or training did the researcher have? | EM completed a qualitative course and special training on Foundation of Qualitative Methodologies, Data Collection and Analysis.  VC & VM are qualitative experts. | Page 5-6 Data Analysis section |
| *Relationship with participants* | | |
| 6. Relationship established  Was a relationship established prior to study commencement? | No | Page 5  Data collection |
| 7. Participant knowledge of the interviewer  What did the participants know about the researcher? e.g. personal goals, reasons for doing the research | Participants were informed on the purpose of the study and understood that it was a research study for EM. | - |
| 8. Interviewer characteristics  What characteristics were reported about the interviewer/facilitator? e.g. Bias, assumptions, reasons and interests in the research topic | EM declared her academic standpoint and participants were made aware that she was not from a medical background. This allowed EM to ask naïve questions and asks for clarifications where needed. | - |
| Domain 2: Study design | | |
| *Theoretical framework* |  |  |
| 9. Methodological orientation and Theory  What methodological orientation was stated to underpin the study? e.g. grounded theory, discourse analysis, ethnography, phenomenology, content analysis | An exploratory, descriptive qualitative study was conducted employing semi-structured interviews and focus group. Data were analysed using the thematic analysis by Braun and Clarke. | See page 4-6 |
| *Participant selection* | | |
| 10. Sampling  How were participants selected? e.g. purposive, convenience, consecutive, snowball | Purposive sampling was used to select participants to capture maximum variation in views and experiences. | See page 4. |
| 11. Method of approach  How were participants approached? e.g. face-to-face, telephone, mail, email | Participants was approached via telephone, email and face-to -face. | See page 5. |
| 12. Sample size  How many participants were in the study? | The final sample comprised of 40 participants (1 focus group n=7 participants and n=33 individual interviews) | See page 5. |
| 13. Non-participation  How many people refused to participate or dropped out? Reasons? | None | - |
| *Setting* | | |
| 14. Setting of data collection  Where was the data collected? e.g. home, clinic, workplace | Focus group were conducted in a meeting room at HMRI. The interviews were conducted either face-to-face in their place of work or over the phone/ online. | See page 5. |
| 15. Presence of non-participants  Was anyone else present besides the participants and researchers? | No one else was present except for interview participants and the researcher. | - |
| 16. Description of sample  What are the important characteristics of the sample? e.g. demographic data, date |  | See page 7-8  See Table 2 |
| *Data collection* |  |  |
| 17. Interview guide  Were questions, prompts, guides provided by the authors? Was it pilot tested? | Interviews were semi-structured using a topic guide. Probes were used to generate further explanation. | See page 5 and Online supplement (see Additional File, S2 Table) |
| 18. Repeat interviews  Were repeat interviews carried out? If yes, how many? | None | See page 5. |
| 19. Audio/visual recording  Did the research use audio or visual recording to collect the data? | Yes, audio recording was used throughout the data collection. | See page 5. |
| 20. Field notes  Were field notes made during and/or after the interview or focus group? | Additional field notes were made after the interviews. | See page 5. |
| 21. Duration What was the duration of the interviews or focus group? | The interview ranged between 13-49 minutes | See page 5. |
| 22. Data saturation  Was data saturation discussed? | Yes, this was discussed in team meetings. | See page 5-6. |
| 23. Transcripts returned  Were transcripts returned to participants for comment and/or correction? | No. However, EM verified the transcript against the sound file for data accuracy. | See page 5-6. |
| *Domain 3: analysis and findings* |  |  |
| *Data analysis* |  |  |
| 24. Number of data coders  How many data coders coded the data? | One | See page 5-6. |
| 25. Description of the coding tree  Did authors provide a description of the coding tree? | Coding described in Data Analysis section. | See page 5-6. |
| 26. Derivation of themes  Were themes identified in advance or derived from the data? | Themes were derived from the data. | See page 5-6. |
| 27. Software  What software, if applicable, was used to manage the data? | Yes, NVivo Pro version 12.0 were used to manage the data. | See page 5-6. |
| 28. Participant checking  Did participants provide feedback on the findings? | No, participants did not provide feedback. | - |
| *Reporting* | | |
| 29. Quotations presented  Were participant quotations presented to illustrate the themes / findings? Was each quotation identified? e.g. participant number | Yes, specific quotations to illustrate key themes are presented in Tables 3,4,5,6 and in the supplementary material Tables S3,S4. All direct quotes are anonymised using a medical profession and participant number. | See pages 12-28.  See Tables 3-5 and online supplement (see Additional Files, S3 Table, S4 Table). |
| 30. Data and findings consistent  Was there consistency between the data presented and the findings? | Yes there was consistency between the data presented and the findings. | See page 12-29. |
| 31. Clarity of major themes  Were major themes clearly presented in the findings? | Yes | See page 12-29. |
| 32. Clarity of minor themes  Is there a description of diverse cases or discussion of minor themes? | Yes, sub-themes of all themes are discussed in the manuscript and reported in full in the supplementary material supplementary along with the manuscript. | See page 12-29. |

S2 Table. Topic Interview Guide.

| **a. Current Practice**  Can you describe to me your roles in severe asthma management?  What do you think is the biggest strength of your current approaches to severe asthma management?  In your experience, what would you say is your biggest problems or issues to your practice?  **b. Knowledge, attitudes, behaviours**  Based on your experiences, what are the most effective approaches to managing patients with severe asthma?  If you had to design or develop an ideal model of care for severe asthma, what would it look like?  **c. Guideline/Referral Process**  Think of a time when you had a situation where you needed to refer a patient for specialist consultation. What was the situation and what led to that decision?  Have there been times when you don’t consider referring severe asthma patients for specialist consultation?  When a patient experience a range of clinical symptoms/comorbidities, how would you strategise your referrals?  What do you think is the best way to implement referral processes?  In your experience, what barriers have you personally encountered in referring severe asthma patient to another specialty or allied health service?  **d. Environment/Resources/Reinforcement**  In your experience, what resources and services do you access to care for severe asthma patients?  What would help you facilitate and implement optimal severe asthma management?  What kind of online resources have you found that help you with your practice?  What education and training would likely to receive to effectively manage severe asthma patients?  **e. Carers**  What experiences have you had in dealing with carers of people with severe asthma?  How do you feel involving carers in decision making around care and treatment? |
| --- |

S3 Table: Barriers to and enablers for optimal management.

| **Theme/sub-theme** | **Illustrative quotations** |
| --- | --- |
| **Barriers to optimal management** | |
| **Patient-related factors** | |
| Attitudes | *The perennial issue of patient’s compliance would be another one.* (RS, P#29) |
| Preference, expectations and beliefs | *I think compliance and that people really want, especially from a speech pathology point of view, I cannot tell you how many people have asked me for a magic pill … I think that’s the biggest issue. People want something that’s a quick fix. They want a medication to fix something that isn’t fixable with a medication. Compliance and the person’s unwillingness to accept that they have control over what’s going on with themselves.* (SP, P#25) |
| Comorbidities | *Some of the concern of course is around obesity and the level—obesity-related disease in respiratory. However, if we don't start doing something about it, it's just going to become a worse problem.* (Nurse, P#2)  *Some of them are easy to treat like vocal cord dysfunction—relatively easy. Some are really difficult, like obesity.* (RS, P#29) |
| Poor knowledge | *I think just shifting the trend from a lot of people, they think Ventolin is the only treatment, where you can get good results with Symbicort by itself, on a ad hoc basis … Poor knowledge.* (Nurse, FG)  *People come to us just believing they just have asthma, and they don’t understand why the medications don’t make it better and it’s because they have asthma co-occurring with other things and the medication for asthma isn’t going to treat upper airway hypersensitivity or vocal cord dysfunction.* (SP, P#25) |
| **System-related factors** | |
| Inadequate funding and incentives | *I think the issues across the board have to be, I think, appropriate funding.* (RS, P#33)  *Room availability is a massive problem.* (Nurse, FG) |
| Workload capacity and complex process | *All the pharmacy applications, I do that as well, so there's no pharmacy support for severe asthma clinic; lots of clerical work involved with it and coordination. So there's no provision for an actual coordinator.* (Nurse, P#2)  *So, my role has expanded to cover asthma education, which is something I’ve, in addition, taken on independently. And look, if—there’s no one dedicated to that role so I—if there’s only one of us here during annual leave, Fridays, sick leave … then our priority as our job description is to see the patients that call at home—that are sick at home. So, if we are out in the community then we’re unable to give asthma education to patients that are in hospital or in ED if there’s only one of us and we’re out doing four visits or five visits a day. So really, there’s no-one dedicated to that role. So, the difficulty of being—visiting patients at home but also providing the education for in patients. I still have four of five patients to see in the community.* (Nurse, P#12) |
| Long waiting list and lack of standardised referral pathway | *The other barrier that is challenging is linking our patients with the respiratory clinics. So, we don’t have rapid access clinic to offer so you might refer to the respiratory CNC and you know what the allied health approach in the clinic, so we don’t have that facility … there’s no clearer referral pathway available to them.* (ED specialist, P#16)  *Once we give medication, it's hard for me to follow up with the customer* [patient] *and chase them. What I tell them, I say, ‘I hope it works for you, if you have any questions come back in and see me. If it works for you, come back and tell me that it works for you, so that I know what to pass on as general feedback for doctors in the future, and other people’.* (Pharmacist, P#3) |
| **Provider-related factors** | |
| Attitudes and communication difficulties between HCPs | *I'd love, at the respiratory clinics at the practice to be able to get partners to come along as well. We don't do that at the moment and the doctors aren't really happy about that.* (Nurse, P#19) |
| Unfamiliarity with new therapies | *I’m actually doing a little bit of reading about this at the moment but with all the new biologics there’s quite—I think this is information that is slowly starting to become—it might be well known to the respiratory specialists but it’s still probably not that well known to those outside of it.* (Nurse, P#12)  *I think there's a lot of health practitioners, nurses and doctors out there who haven't kept up to date with what's happening with the treatment of asthma, and they're heads are back in the way things used to be.* (Nurse, P#22)  *general practice is a huge area of need, in terms of understanding about what factors could be contributing to the symptoms that may be also caused by severe asthma. So, GPs don’t have much time, and they don’t have much expertise in managing except in a fairly simplistic way.* (RS, P#14) |
| Resource constraints | *Again, our pharmacy's not funded for outpatients at all, so there is no outpatient pharmacy whatsoever—our pharmacists, unless it's being funded specifically for a role. there is very little social work for inpatients, let alone outpatients.* (Nurse, P#2)  *Compared to other diseases, the challenges that we face in terms of getting access to, well, all of these disciplines, to have time—to make time at the same time every week or every month, or—it’s a bit challenging that way.* (RS, P#28)  *The lack of the specialised staff can just delay the patient’s care.* (RS, advanced trainee, P#1)  *In private practice, we don't have the ability to link into a clinical nurse educator, and it's the same at the private hospitals. There are respiratory nurses on the ward, but there's no outpatient role for a clinical nurse with expertise in asthma.* (RS, P#30)  *One of the things I find that very challenging is we find in a situation we’re we try to get them home, we lack bed for patient … we’re not able to access the pharmacist in the emergency department, it would really be vital for having them in ED.* (ED specialist, P#16) |
| **Theme/sub-theme** | **Illustrative quotations** |
| **Enablers for optimal management** | |
| **Knowledge, skills and experience** | |
| Competence, confidence and expert roles | *I've got a good understanding of the evidence behind treatments. I'm able to assimilate new information rapidly and instigate or translate whatever the best practice and treatments are into the service we currently are able to provide and within the borders of what we can do here in our metropolitan centre severe asthma clinic.* (Nurse, P#3)  *My strength is I actually get to see the script before it goes—before the medication goes to the customer. I check and see what the doctor has prescribed, I have the opportunity to go to the patient and discuss their asthma action plan and to see what happened for them to have severe asthma and educate them and tell them how to take their medication appropriately.* (Pharmacist, #P3) |
| **Team-based and multidisciplinary approach** | |
| Collaborative and systematic approach to care | *It’s not just nurses working separately, allied health separately. Our physicians really much value their team who they work with and realise how important it is to have absolutely everyone on that team. So, I think we already have that magic sort of collaboration there and I guess magic wand of continued collaboration I think is important.* (SP, P#25)  *The strengths are the fact that it’s a multidisciplinary clinic, that the approach is based on evidence and on evidence-based practical tools, and that we have two-way traffic between research and clinical practice.* (RS, P#14)  *Having a team-based approach is really important. So, having the staff that we have in the emergency department, who are also familiar with the protocols is fantastic, because we're all on the same page right from the very beginning.* (ED specialist, P#10) |
| **Creating and supporting environment for person-centred care** | |
| Partnerships between HCPs, patients and their families | *I think it’s critical that you have another family member involved if you can in the severe asthma management because it just makes the whole process of people understanding and using asthma plans and understanding the intent of treatment et cetera.* (RS, P#29)  *If they're in the clinic, I make sure that they hear the same information that the patient hears. We have a discussion, often a three-way discussion, if we're going to embark on a new treatment strategy what that’s going to look like. Are there any expectations on the carer in terms of what they might need to do, all the pros and cons of going down one treatment path versus another treatment path?* (RS, P#23)  *But we do try to involve carers at the bedside as much as possible, because they're the ones who are going to be looking after their patients—their people. So, we invite them to come in and be there with them, and it's often reassuring for the patient who is unable to breathe or talk or give any indication about their wants or needs or desires, and their carer knows them.* (ED specialist, P#13) |
| **Tools and resources** | |
| Online and practical resources | *we make use of the Severe Asthma Toolkit obviously. So that gets used a lot. Yeah, and we use that to point—it's often a great resource for educating patients, educating other healthcare providers, that sort of thing.* (RS, P#23)  *I'm a big fan of the online tools, the Severe Asthma Toolbox and things.* (RS, P#30)  *Yes, the GINA guidelines are very important, or the GINA strategy, has a lot of information there including severe asthma. I think their most recent algorithms are very helpful that have clinical pathways which are useful globally, need to adapt that locally.* (RS, P#15)  *Yes, I do use online resources. So, for children I'll use the Royal Children's Hospital guidelines just to double check doses. Especially for intravenous infusions. So, I'll use RCH for children. For adults, I'll use our intranet guidelines, like ICU guidelines to double check infusions.* (ED specialist, P#10) |
| **Referral strategies** | |
| Creating effective referrals | *Well … if there's comorbidities, like diabetes, or an existing lung problem, then definitely I do the referral right away* (GP, P#31)  *Depending on their other problems. I think I would refer. But sometimes, if the magnitude of the problem is not that much, you want to concentrate on what you are doing first before you refer it on. So, it depends on the other comorbid problems and their magnitude.* (RS, P#27)  *It's a matter of working out which of the comorbidities are making the biggest impact on their symptoms. So, if you think that sinus disease or nasal polyps is really a key issue, well, then I'd initiate an ENT referral.* (RS, P#23) |
| Access | *So, where I suspect that as a comorbidity or as an alternative cause for the symptoms, what I do in that case is I find out where the patient lives, which area health service they fall into and then I give them a referral to that particular service provider.* (RS, P#14)  *I do refer quite a lot to speech pathology, and I use the public speech pathology clinic because they have an interest in vocal cord dysfunction.* (RS, P#30) |
| **Approach to personalised care** | |
| Multidisciplinary team care | *Well, ideally, I would say a multidisciplinary model in a severe asthma clinic in the hospital would work really well.* (RS, P#28)  *I think something that's a lot more open and more responsive would be really quite good. I think having a one-stop shop which is available for people to come would be good.* (RS, P#26)  *Obviously multidisciplinary care is important in absolutely everything we do and I think that we are really fortunate that in the current time that we have such good multidisciplinary care.* (SP, P#25)  *Just that holistic team, multidisciplinary team approach to the care of the person, I suppose, with their goals and their ideas and whatever at the centre.* (Nurse, P#19) |
| **Referral pathways** | |
| Referral tools and pathways | *Whether there's like a safety net you can—yeah … Whether we just have, like as a checklist scenario, it's a potential thing. Whether it's a physical checklist or an electronic one or even just a mental checklist.* (RS, advanced trainee, P#1)  *Just like, clear and concise pathways, really—and for all phases of asthma. So, because once they’re stabilised* [or have] *been stabilised in ED, and they get transferred to the unit, it doesn’t mean that—you know, they’re not going to deteriorate, or anything. So, just ongoing education, really. How to manage them* [in all settings]*, like how to manage someone with severe asthma in all settings and what to look for if they are deteriorating, or they’re about to have another onset of an acute …* (Nurse, P#11) |
| **Management plan** | |
|  | *Well, one would think the GP’s going to refer through to us if they’ve concerns, otherwise we’re going to pick them up through admission or ICU.* (Nurse, FG) |
| **Elements to optimal management** | |
| Sufficient knowledge and information | *I know in the states they have these respiratory technicians, or respiratory therapists who are experts in managing non-invasive ventilation in the ventilator. Sometimes that's an area of expertise that may be slightly lacking in myself and my colleagues in the emergency department. So, maybe having either more training for ourselves, or experts that are available to help work the machines might be helpful.* (ED specialist, P#10)  *In my setting, probably education around triage management. Having said that, for severe asthma, it's pretty straightforward. If somebody is in respiratory distress, it's a pretty easy decision to make. But always triage—education is always important around identification of asthma. And any kind of upcoming technologies and things like that, so research in terms of what really works for asthma. That would help me too. So, any kind of updated research on management of asthma is always going to help.* (Nurse, P#24) |
| Adequate resources to deliver equitable care | *I think access to an MDT would be a really important thing for a lot of places, and that doesn't have to be in person. That has to—that could be sort of phone or telehealth.* (RS, advanced trainee, P#1) |
| Resources for patients and their families | *I think carers do understand the problems if they’re given more. Like, for instance, some material to the carers, I think they will understand the disease a little bit better.* (RS, P#27) |
| Improving quality care | *So at the moment it’s just face-to-face individual education. But I suppose—look, really, I mean there’s no reason why you couldn’t do that in a pulmonary rehabilitation style education where you're educating a room of people rather than trying to catch people before they're out the door which is often maybe not dedicating the right value and time to patients depending on the other things that have to be done.* (Nurse, P#12)  *I guess if someone lived independently with minimal family support or something, but you’d want some type of safety plan or a plan in place. Someone to know—if they wear an ID bracelet or something.* (Nurse, P#12) |
| Resource-sharing | *Probably we should have to have a support person ... I know F2, they have a CNC or CNS, a respiratory nurse, we need that one in our ward also. There's not even—they don't need to stay there but at least to guide us and continuously give us education.* (Nurse, P#18)  *By ensuring that we’re having interdepartmental meetings with more sharing of resources, then I think it would have hopefully a good effect in terms of ensuring that communication with those first line carers and primary care centres would be optimised as well.* (ED specialist, P#16) |

*Note*. RS = respiratory specialist; P# = participant no.; SP = speech pathologist; FG = focus group; ED = emergency department; CNC = clinical nurse consultant; HCP = healthcare professional; GINA = Global Initiative for Asthma; RCH = Royal Children’s Hospital; ICU =intensive care unit; GP = general practitioner; MDT = multidisciplinary team; CNS = clinical nurse specialist.

S4 Table. Desired model of care.

| **Theme** | **Sub-themes** | **Illustrative Quotes** |
| --- | --- | --- |
| Desired model of care | ***Approach to personalised care*** | |
|  | Multidisciplinary team care | *Well, ideally, I would say a multidisciplinary model in a severe asthma clinic in the hospital would work really well. (RS, P#28)*  *I think something that's a lot more open and more responsive would be really quite good. I think having a one-stop shop which is available for people to come would be good. (RS, P#26)*  *Obviously multidisciplinary care is important in absolutely everything we do and I think that we are really fortunate that in the current time that we have such good multidisciplinary care. (SP, P#25)*  *Just that holistic team, multi-disciplinary team approach to the care of the person, I suppose, with their goals and their ideas and whatever at the centre. (Nurse, P#19)* |
|  | ***Referral pathways*** | |
|  | Referral tools and pathways | *Whether there's like a safety net you can – yeah… Whether we just have, like as a checklist scenario, it's a potential thing. Whether it's a physical checklist or an electronic one or even just a mental checklist. (RS, Advance Trainee, P#1)*  *Just like, clear and concise pathways, really – and for all phases of asthma. So, because once they’re stabilised [or have] been stabilised in ED, and they get transferred to the unit, it doesn’t mean that – you know, they’re not going to deteriorate, or anything. So, just ongoing education, really. How to manage them [in all settings], like how to manage someone with severe asthma in all settings and what to look for if they are deteriorating or they’re about to have another onset of an acute… (Nurse, P#11)* |
|  | Management plan |  |
|  |  | *Well, one would think the GP’s going to refer through to us if they’ve concerns, otherwise we’re going to pick them up through admission or ICU. (Nurse, FG)* |
|  | ***Elements to optimal management*** | |
|  | Sufficient knowledge and information | *I know in the States they have these respiratory technicians, or respiratory therapists who are experts in managing non-invasive ventilation in the ventilator. Sometimes that's an area of expertise that may be slightly lacking in myself and my colleagues in the emergency department. So, maybe having either more training for ourselves, or experts that are available to help work the machines might be helpful. (ED Specialist, P#10)*  *In my setting, probably education around triage management. Having said that, for severe asthma, it's pretty straightforward. If somebody is in respiratory distress, it's a pretty easy decision to make. But always triage - education is always important around identification of asthma. And any kind of upcoming technologies and things like that, so research in terms of what really works for asthma. That would help me too. So, any kind of updated research on management of asthma is always going to help. (Nurse, P#24)* |
|  | Adequate resources to deliver equitable care | *I think access to an MDT would be a really important thing for a lot of places and that doesn't have to be in person. That has to - that could be sort of phone or tele-health. (RS, Advance Trainee, P#1)* |
|  | Resources for patients and their families | *I think carers do understand the problems if they’re given more. Like, for instance, some material to the carers, I think they will understand the disease a little bit better. (RS, P#27)* |
|  | Improving quality care | *So at the moment it’s just face to face individual education. But I suppose – look, really, I mean there’s no reason why you couldn’t do that in a pulmonary rehabilitation style education where you're educating a room of people rather than trying to catch people before they're out the door which is often maybe not dedicating the right value and time to patients depending on the other things that have to be done. (Nurse, P#12)*  *I guess if someone lived independently with minimal family support or something, but you’d want some type of safety plan or a plan in place. Someone to know – if they wear an ID bracelet or something. (Nurse, P#12)* |
|  | Resource sharing | *Probably we should have to have a support person... I know F2, they have a CNC or CNS, a respiratory nurse, we need that one in our ward also. There's not even - they don't need to stay there but at least to guide us and continuously give us education. (Nurse, P#18)*  *By ensuring that we’re having interdepartmental meetings with more sharing of resources, then I think it would have hopefully a good effect in terms of ensuring that communication with those first line carers and primary care centres would be optimised as well. (ED-Specialist, P#16)* |

*Note*. RS = respiratory specialist; P# = participant no.; SP = speech pathologist; FG = focus group; ED = emergency department; CNC = clinical nurse consultant;

HCP = healthcare professional; GINA = Global Initiative for Asthma; RCH = Royal Children’s Hospital; ICU =intensive care unit; GP = general practitioner;

MDT = multidisciplinary team; CNS = clinical nurse specialist.
